# Implementing COVID-19 response within the context of the broader health system: a proposed framework for Africa´s policy makers

**DOI:** 10.11604/pamj.supp.2020.35.2.23574

**Published:** 2020-05-22

**Authors:** Abdu Abdullahi Adamu, Rabiu Ibrahim Jalo, Abdulkarim Ibrahim Dahiru, Charles Shey Wiysonge

**Affiliations:** 1 Cochrane South Africa, South African Medical Research Council, Tygerberg, South Africa; 2 Division of Epidemiology and Biostatistics, Department of Global Health, Faculty of Medicine and Health Sciences, Stellenbosch University, Cape Town, South Africa; 3 Department of Community Medicine, Bayero University/Aminu Kano Teaching Hospital, Zaria Road, Kano State, Nigeria; 4 Department of Health Planning, Research and Statistics, Nassarawa State Ministry of Health, Lafia, Nassarawa State, Nigeria; 5 School of Public Health and Family Medicine, University of Cape Town, Cape Town, South Africa

**Keywords:** COVID-19, Africa, health system

## Abstract

Heads of government in Africa responded to the COVID-19 pandemic by setting up high-level task forces at continental and national levels to coordinate preparedness and response strategies, in a bid to mitigate the spread of this virus on the continent. However, the current strategy at both continental and national levels are narrowly focused on COVID-19 and this is not sustainable. This is because Africa has a high burden of communicable and non-communicable diseases and sustaining access to essential life-saving health services is also critical during this pandemic. Therefore, we call for a more holistic health systems-based model for COVID-19 outbreak response. We recommend that response strategies should be transitioned from vertical isolated programmes to a broad-based “time-bound” integrated health system intervention that links with existing health programmes as well as other government and non-governmental sectors.

## Commentary

On account of the growing number of coronavirus disease 2019 (COVID-19) cases and reported international spread, as well as the potential risk that the new disease could pose to countries with weaker health systems, the World Health Organization (WHO) on 30th January 2020, declared it as a public health emergency of international concern (PHEIC) [1]. Accordingly, heads of government in African countries responded by setting up high-level task forces at continental and national levels to coordinate preparedness and response, in a bid to mitigate the spread of this disease on the continent [2]. In an emergency meeting of the continent´s ministers of health on February 2020, policies such as quarantine, movement restriction, cross border cooperation and private sector engagement were proposed [3]. The African Union has developed a continental strategy for coronavirus disease 2019 (COVID-19) outbreak, to galvanize collaboration between countries and enhance technical synergies for surveillance, infection prevention and control, laboratory diagnosis, risk communication, clinical management, port of entry screening and supply chain management [2].

What is omitted in this strategy, which has already been adopted by countries, is the linkage and interaction between the COVID-19 outbreak response and the broader health systems. COVID-19 can have a devastating effect on healthcare resources, as already seen in other places [4]. People with moderate and severe disease require medical care and an exponential growth in cases can overwhelm the health system [5]. Nonetheless, Africa´s health system is already stretched. The continent has the highest number of people living with human immunodeficiency virus (HIV) in the world [6]. In addition, it also has the highest burden of tuberculosis (TB) and TB-HIV co-infection, with a significant proportion of global drug resistant TB [7]. Furthermore, while the annual birth cohort is high, routine immunization coverage is suboptimal and stagnated [8]. Consequently, health facilities have to regularly attend to a high number of children with vaccine-preventable diseases. So, a surge in COVID-19 caseload will compete with these existing health needs.

If left unaddressed, people can potentially face a significant reduction in access to life-saving health services like immunization, HIV care and treatment, directly observed TB treatment and community programmatic management of drug-resistant TB (c-PMDT) among others. These far reaching implications necessitates a COVID-19 response strategy that is conceived bearing in mind the prevailing complexity of health problems on the continent. Unfortunately, the current strategy in Africa leans towards the outbreak narrative´ [9] and this alone might not be sufficient. The focus is overtly on controlling the epidemic and restoring the status quo, with less consideration for existing health problems and attending social dynamics. This is not surprising as epidemic governance literature suggests that in times of outbreaks, most leaders tend towards control as a style of action together with the temporality of change perceived as short-lived shocks [9]. This can results in stability which is good in the short term but does not lead to resilience, robustness, or durability which are elements that leads to a strengthened system [9].

**Figure 1:**
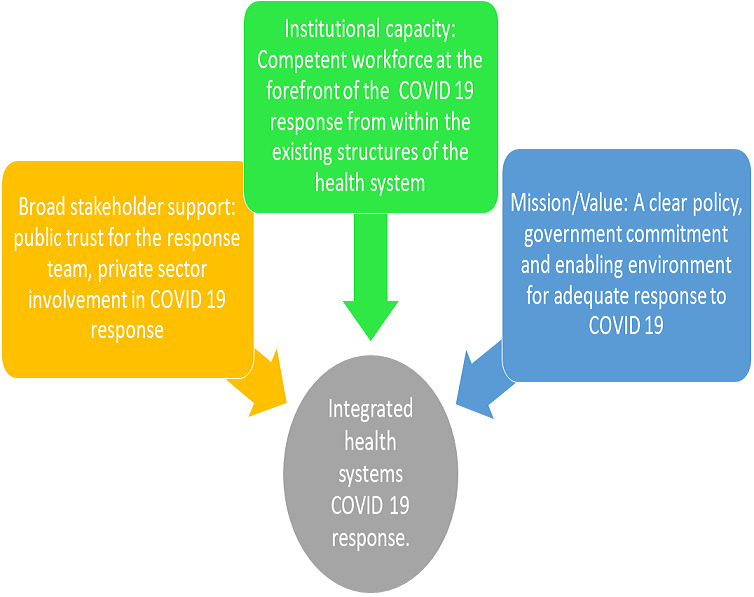
Adaptation of Moore’s strategic triangle to a systems strengthening COVID-19 response

**Figure 2:**
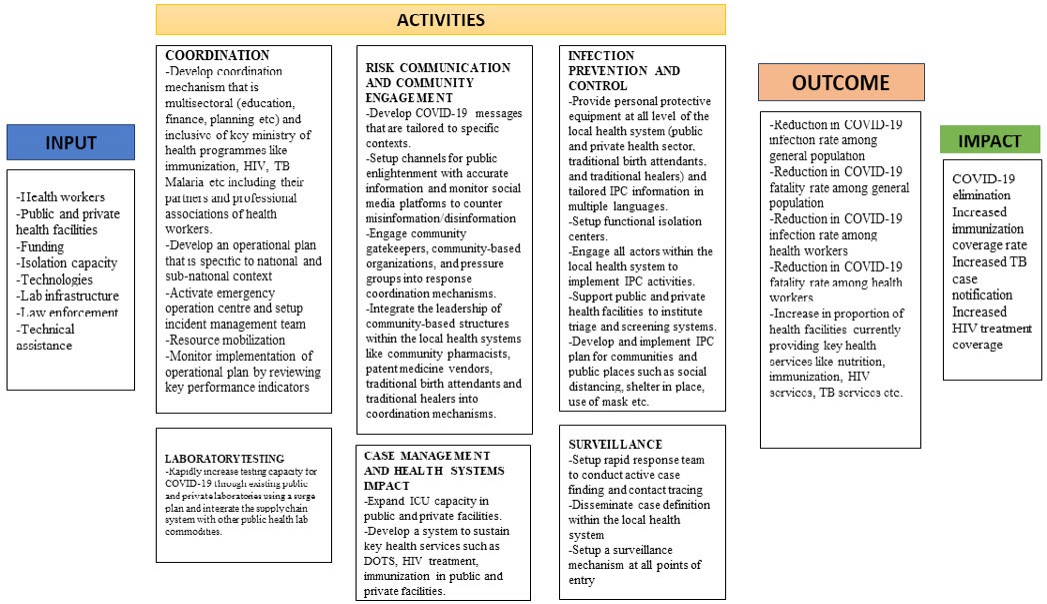
Logic framework for implementing COVID-19 response within the broader health systems context

We therefore recommend that the strategy at continental and national level should be revised [2] and a more holistic health systems-based model for COVID-19 outbreak response adopted. The current response efforts should be transitioned from vertical isolated programmes to a broad-based “time-bound” integrated health system intervention that links with existing health programmes as well as other government and non-governmental sectors. To achieve this integration, policy makers can leverage on the proposed model shown in Figure 1 which draws on Moore´s strategic triangle [10]. Moore theorized that for success to occur, a clear mission must be accompanied by sound operational capacity and far reaching broad stakeholder support [10]. This programmatic framework further maps out the logic model (Figure 2) into three key strategic areas of focus. The first being a clear mission by the government with sound policies and clear focus. The next is to have adequate operational capacity to implement these policies and actions within the existing health systems using already existing inputs and processes.

The final strategy is to build public trust and ensure broad stakeholder support especially the private sector, donors, implementing partners, professional bodies and civil societies. Hence, the existing high-level task forces should maintain a strategic role, but operational tasks as contained in the technical pillars for COVID-19 control should be implemented through existing health programmes and structures. For example, the different levels of the hospital system can be mobilized to implement rapid response to support active COVID-19 case finding within their catchment areas as part of regular services. Also, to expedite testing, COVID-19 laboratory services can be decentralized and integrated into existing public health laboratory platforms. Furthermore, public health programmes like the national tuberculosis programmes (NTPs) and expanded programmes on immunization (EPIs) have service delivery mechanisms that should be empowered to sustain services delivery within the context of COVID-19. This way, sustaining access to health care for existing health needs during the pandemic can become positioned as one of the core goals of the response.

The guiding principles for this proposed framework include, inter alia, people-centeredness, community-driven approach and strengthening of mixed health systems. In this context, people-centered COVID-19 response will involve deploying innovative strategies to sustain access to health services that people routinely use like childhood immunization, HIV treatment and other services during the pandemic. The response should be community-driven; ensuring that communities participate in the planning and operationalization of COVID-19 control strategies, sustaining essential community health services like immunization outreach and community PMDT and tackling social determinants that may affect health care utilization during the pandemic or compliance with infection control strategies. Strengthening mixed health systems require robust engagement of private health sector actors as well as community-based health structures such as patent medicine vendors, traditional birth attendants and community health workers. In short, the entire local health system should be working synchronously to achieve two goals: control COVID-19 and sustain health services.

If COVID-19 control activities become fully integrated with the health system, then in addition to COVID-19 outcomes, other outcomes that track health system functionality should be monitored, reported, and acted upon during the pandemic. An example of such non-COVID-19 outcomes could be the proportion of health facilities providing immunization services. When compared to the pre-outbreak period, if a decline is detected, then immediate action can be instituted to rectify it. Similarly, the overall impact of the COVID-19 response should incorporate key targets from other disease control programmes. In conclusion, the multidimensional nature of Africa´s health needs vis-à-vis the disruptive capacity of COVID-19 on the health system necessitates a shift from disease-focused response framework, to a broader health systems approach. This can minimize the interruption of health services at national and sub-national level during the pandemic and improve the resilience of the health system. Although we proposed a framework to spur action, it is by no means prescriptive and can be adapted to specific country contexts.

## Competing interests

The author declares no competing interests.
